# Elevated Creatinine Kinase in Peripheral Neuropathy Is Associated With Muscle Cramping

**DOI:** 10.3389/fneur.2021.613599

**Published:** 2021-02-04

**Authors:** Allison Jordan, Arun Nagaraj, J. Chad Hoyle, Amro Maher Stino, W. David Arnold, Bakri Elsheikh

**Affiliations:** Department of Neurology, The Ohio State University Wexner Medical Center, Columbus, OH, United States

**Keywords:** cramping, muscle enzyme, CK, neuropathy, peripheral neuropathy

## Abstract

**Introduction:** Serum Creatinine Kinase (CK) is a non-specific marker of muscle damage. There has been limited investigation of the association between peripheral neuropathy and CK elevation (hyperCKemia).

**Methods:** We performed a chart review to investigate the CK level in peripheral neuropathies. Demographics, clinical history, physical exam, electrodiagnostic data, CK level, statin use, etiology of neuropathy, and concomitant neuromuscular disorders were recorded. HyperCKemia was defined using our laboratory cutoff values of >180 U/L (women) and >220 U/L (men).

**Results:** We identified 450 patients with peripheral neuropathy who had CK testing, 92 (20.4%) of whom had hyperCKemia. Sixty-one of those patients (13.5% of the total figure) had a concomitant etiology that could explain the CK elevation. Thirty-one patients (6.9%) had no other identifiable etiology for their hyperCKemia beyond the neuropathy. The average CK level in the latter cohort with hyperCKemia was 376 U/L (women: 312 U/L; men: 444 U/L). The frequency of cramping was greater in patients with elevated vs. normal CK (*p* < 0.0001).

**Discussion:** HyperCKemia can occur in patients with peripheral neuropathy and appears to associate with cramping.

## Introduction

Creatinine Kinase (CK) is an intramuscular enzyme that catalyzes the combination of creatinine with adenosine triphosphate (ATP) to form phosphocreatine. CK, along with other enzymes such as lactate dehydrogenase, aldolase, myoglobin, and troponin, are nonspecific, albeit useful, markers of muscle integrity, as they are released into the bloodstream during muscle stress and injury (1). Clinicians often struggle to identify an etiology for hyperCKemia, which is most often attributed to a myopathic process. Nevertheless, hyperCKemia has been described in about half of patients with amyotrophic lateral sclerosis (ALS) and has been identified as a possible prognostic factor in the disease (2). HyperCKemia also occurs, to a lesser extent, in acquired neuropathic processes, such as Chronic Inflammatory Demyelinating Polyradiculoneuropathy (CIDP) (3), Guillain-Barre syndrome (GBS) (4), and multifocal motor neuropathy with conduction block (MMN) (5). Rarely, hyperCKemia associates with Charcot-Marie-tooth (CMT) disease (6, 7). Such prior findings suggest that peripheral neuropathy may associate with hyperCKemia, although this association is not well defined in the literature. We conducted this study to evaluate the frequency of hyperCKemia in peripheral neuropathy and explore potential etiologies.

## Methods

This retrospective study was approved by the institutional review board at The Ohio State University Wexner Medical Center. A chart review was conducted of patients 18 years of age or older seen at The Ohio State University Wexner Medical Center between May 2013 and May 2018. Patients needed to carry a diagnosis of peripheral neuropathy, as clinically determined and documented by a neurological examination conducted by a neuromuscular neurologist and to have also undergone CK testing. Large fiber peripheral neuropathy was diagnosed based on abnormalities on vibratory, proprioceptive, and distal motor deficits on examination. Small fiber peripheral neuropathy was diagnosed by suggestive symptoms and/or distal pinprick sensory loss, in the absence of large fiber findings. Demographic data, clinical history, physical examination findings, EMG data, CK level, statin use, etiology of peripheral neuropathy, and concomitant neuromuscular disorders were documented. We defined hyperCKemia as CK > 180 U/L in females and CK > 220 U/L in males based on our institutional normative values. We excluded African American patients from the above definition of hyperCKemia. It has been well established that African American patients have higher mean baseline CK levels than Caucasian patients, which reflects a mean value well above our institutional laboratory cut off (8). We performed descriptive analyses to document findings and the Mann–Whitney U-test for group comparison. A *p*-value < 0.05 was considered significant.

## Results

**Figure 1:**
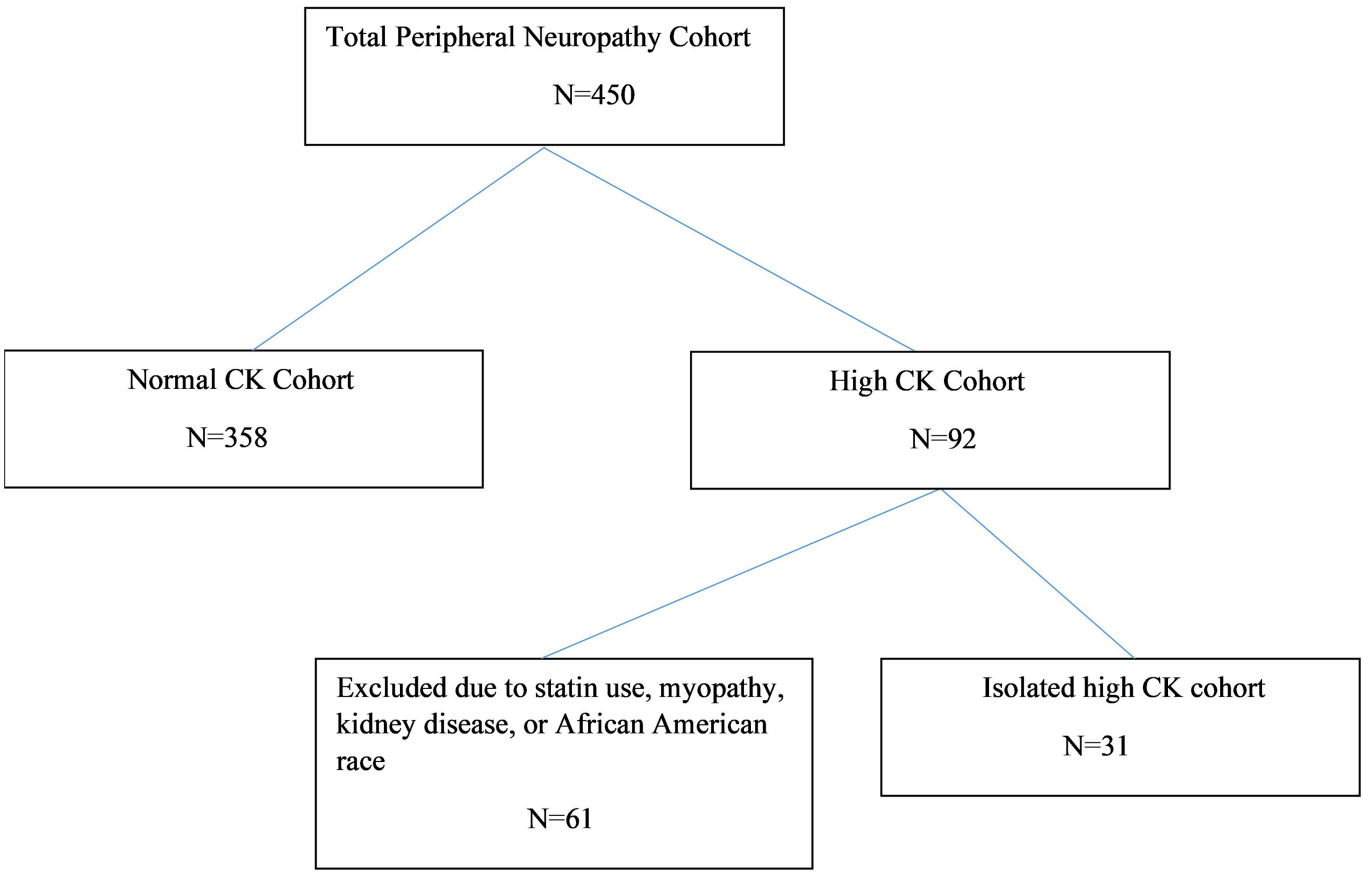
Peripheral neuropathy patient disposition diagram.

We identified 450 patients with confirmed peripheral neuropathy and CK testing, 92 (20.4%) of whom had hyperCKemia. Of the 92 patients with hyperCKemia and peripheral neuropathy, 61 had possible etiologies or explanations, including myopathy, motor neuron disease, statin use, or chronic kidney disease. Patients of African American ethnicity were excluded on the basis of differences in baseline mean CK. Thirty-one patients, representing 6.9% of our total peripheral neuropathy cohort of 450, had no other identifiable etiology for hyperCKemia and were further analyzed (see Figure 1).

The mean age of the patients with peripheral neuropathy and normal CK (*n* = 358) was 57 ± 14 years. One hundred seventy-three (48.3%) were men (see Table 1A). Three hundred one patients (84.1%) identified as Caucasian, 39 (10.9%) as African American/African, 5 (1.4%) as Asian, 3 (0.8%) as American Indian, and 10 (2.8%) identified as being of other ethnicity or multi-ethnic. The mean age of the patients with peripheral neuropathy and hyperCKemia but no alternate identifiable etiology was 59 years ± 14.32, and 15 (48%) were men (see Table 1A). Of the patients with peripheral neuropathy and hyperCKemia, 29 patients (93.5%) identified as Caucasian, with one individual identifying as American Indian, and another identifying as multi-ethnic. Using our institutional CK cut-off values, 9% of the 330 Caucasian patients and 28% of the 54 African American patients with peripheral neuropathy had hyperCKemia (see Table 1B).

**Table 1A:** Demographic data of peripheral neuropathy patients.

**Characteristics**	**Normal CK cohort**	**Elevated CK cohort**	
	***n* = 358**	***n* = 31**	***P*-value**
Men	173 (48.3%)	15 (48%)	>0.999
Women	185 (51.7%)	16 (52%)	
Age (mean ± SD)	57 years ± 14.08	59 years ± 14.32	0.274

**Table 1B:** CK data stratified based on race.

**Race**	**Normal CK cohort**	**Elevated CK cohort**	**Total number**
Caucasian	301 (91%)	29 (9%)	330
African American	39 (72%)	*	54
Asian	5 (100%)	0 (0%)	5
American Indian	3 (75%)	1 (25%)	4
Other or more than one race	10 (91%)	1 (9%)	11

* *15 (28%) of all patients of African American race were excluded from analysis of the high CK group*.

The mean CK in patients with peripheral neuropathy without additional etiologies was 376.5 ± 229.5 IU/L (Range: 1,051–193 U/L). All of the patients in this group underwent standard EMG testing. Quantitative EMG studies were not performed. Patients with peripheral neuropathy and hyperCKemia were three-fold more likely to report cramping. A total of 19 patients (61.2%) in the hyperCKemia group reported cramping, compared with 71 (19.8%) patients in the normal CK group (*p* < 0.0001) (see Table 2A). On separate sub-group analysis of African American patients whose CK values exceeded our laboratory cut off, none of the CK values reached a significance of three times upper limit of normal (range 292–825 U/L). In our patients with peripheral neuropathy, hyperCKemia was twice as likely to occur in patients with large fiber peripheral neuropathy as compared to those with small fiber peripheral neuropathy (see Table 2B).

**Table 2A:** Peripheral neuropathy cohort characteristics.

**Characteristics**	**Normal CK cohort**	**Elevated CK cohort**	***P* value**
	***n* = 358**	***n* = 31**	
Total cohort CK U/L (means ± SD)	84.2 ± 50.6	376.5 ± 229.5	
Female CK U/L (means ± SD)	71.0 ± 41.9	312.9 ± 173.4	
Male CK U/L (means ± SD)	98.3 ± 55.3	444.4 ± 266.5	
Associated cramping	71 (19.8%)	19 (61.2%)	<0.0001

**Table 2B:** CK data stratified based on neuropathy type and etiology.

**Neuropathy type**	**Normal CK cohort**	**Elevated CK cohort**	**Total number**
**Large fiber neuropathy**	259 (91%)	27 (9%)	286
Idiopathic	88 (91%)	9 (9%)	97
Hereditary	15 (65%)	8 (35%)	23
Immune	29 (85%)	5 (15%)	34
Glucose tolerance	62 (95%)	3 (5%)	65
Chemotherapy	17 (89%)	2 (11%)	19
Multifactorial/other	48 (100%)		48
**Small fiber neuropathy**	99 (96%)	4 (4 %)	103

Among patients with large fiber peripheral neuropathy and hyperCKemia, common etiologies included hereditary (35%), immune-mediated (15%), chemotherapy-induced (11%), and diabetic/prediabetic (5%) peripheral neuropathies. Based on the EMG findings, the neuropathy was axonal in twenty-two, demyelinating in three, and mixed in one.

## Discussion

Our results show that hyperCKemia is a common finding in patients with peripheral neuropathy. The European Federation of Neurologic Societies (EFNS) published guidelines on the approach to pauci- or asymptomatic hyperCKemia in 2010 (9). These guidelines define significant CK elevation in such patients as being 1.5 times the upper limit of normal, and affirm that race and gender should be factored. Based on these guidelines, cut points for females would be a CK value of 325 U/L and for males would be 504 U/L. Our mean CK values fell close to this range (see Table 2), and a significant percentage of our patients [9 patients (29%) with isolated idiopathic peripheral neuropathy] met or exceeded this cutoff value.

Regarding the variation in hyperCKemia prevalence by peripheral neuropathy etiology, we found the most frequent to be hereditary neuropathy (35%), and the lowest frequency among those with peripheral neuropathy to be glucose intolerance (9%). It is interesting to note that four of our patients with hyperCKemia-associated peripheral neuropathy were small fiber, which is indeed surprising. While an explanation is not clear, some reports describe a high prevalence of cramping in small fiber neuropathy (10). With regards to hyperCKemia occurrence in peripheral neuropathy as compared to other non-myopathic neuromuscular disorders, data exists showing a prevalence of hyperCKemia of 43% in ALS (11). While our study establishes an association between idiopathic hyperCKemia and peripheral neuropathy, we cannot identify a cutoff value that would reproducibly distinguish peripheral neuropathy from other etiologies. However, in 97% of the times the CK value was under 1,000 U/L.

A normal EMG has a strong negative predictive value (74–80%) in excluding myopathy on muscle biopsy in patients with asymptomatic or mildly symptomatic hyperCKemia (9). What is less clear in the current literature is the diagnostic utility and clinical benefit of performing a muscle biopsy in a patient with hyperCKemia and an abnormal EMG that shows only neurogenic changes. Joy et al. looked at muscle biopsy results in subjects with myopathic EMGs. They found EMG to have 93% specificity and 100% sensitivity in predicting an abnormal muscle biopsy result (12). This study was small, however, with only 19 study subjects. A larger study of 100 patients by Prelle et al. showed less favorable sensitivity for EMG in predicting abnormal muscle biopsy (51%) (13). This study defined abnormal EMG as including both myopathic and neurogenic findings, but did not clarify if muscle biopsy findings in patients with neurogenic EMG findings showed superimposed myopathy that may have not been detected on needle examination. In our cohort of 31 patients with isolated peripheral neuropathy and hyperCKemia, two patients underwent muscle biopsies, which showed isolated neurogenic changes without associated myopathic changes. We should note that, in our study, the majority has axonal neuropathy on nerve conduction studies. However, we did not quantitatively compare the severity or degree of positive sharp waves or fibrillation potentials on needle EMG in peripheral neuropathy patients with and without hyperCKemia.

Our study findings further support the notion that peripheral neuropathy is associated with hyperCKemia, and that this association can occur in common peripheral neuropathies such as diabetic neuropathy. Our results suggest that patients with hyperCKemia and peripheral neuropathy may not require further work-up with a muscle biopsy. Care must be taken, however, to always evaluate for certain disorders that may feature combined nerve and muscle involvement (neuromyopathy), such as amyloid, critical illness weakness, mitochondrial neuropathies with multi-system involvement, and even reported cases of inclusion body myositis, namely Valosin containing protein disease (14) Interestingly, epidemiological studies reported that high CK values might occur in the general population (15). These studies included patients with variables such as chronic kidney disease and statin use that could be associated with asymptomatic hyperCKemia. On the other hand, our neuropathy population is a restricted cohort that accounted for these variables, thus strengthening the conclusion that hyperCKemia is a unique finding that can be seen with isolated neuropathy.

One of the most interesting results of this study was the increased prevalence of cramping in peripheral neuropathy patients with hyperCKemia as compared to those with normal CK. Of note, we did not specifically capture electrophysiologic markers of cramping on EMG in this study. Muscle cramping has a well-known association with peripheral neuropathy (10, 16, 17), and has been hypothesized to occur due to abnormal spontaneous firing of terminal motor axons (18). Therefore, it could be hypothesized that in a certain subset of patients, damaged distal nerve terminals trigger irregular axonal firing and muscle contraction, causing mild muscle membrane breakdown and leakage of CK into the bloodstream. Why only a subset of patients develops cramping and hyperCKemia is unclear, as all peripheral neuropathy patients should conceivably be at equal risk of cramping. With regards to the association between cramping and neuropathy etiology, diabetes has been at the center of previous research. A 2014 study demonstrated an increased age-adjusted prevalence of cramping in type 2 but not type 1 diabetic patients as compared with healthy controls (19), and found neuropathy to be the single most impactful predictor for the development of cramps in diabetic patients. A separate Italian study on the use of Botulinum toxin in diabetic patients found a cramp prevalence of 57.8%, although authors did not distinguish type 1 from type 2 diabetic patients (20). The prevalence of cramping in our study was lower as compared with prior studies, and this may be related to the fact that we did not quantify cramping as a complaint during clinical evaluation. Our study identified only a 5% prevalence of hyperCKemia among the 65 diabetic neuropathy patients included, and thus, it is possible that patients with more mild cramping might have not have been identified. While this figure seems quite low, it is worth noting that many patients with normal CK often report cramping, and cramping prevalence was not specifically assessed for our diabetic subgroup of patients. This could suggest a threshold of cramping severity that is required for induction of CK elevation, but we did not quantify cramping severity, which is not routinely quantified as part of our medical records. Thus, this deserves attention in future studies. It is interesting to note that other peripheral neurogenic etiologies that are strongly associated with hyperCKemia, such as ALS and MMN, can also have prominent cramping (21). It is possible that a higher percentage of peripheral neuropathies are associated with mild elevation of CK at some point in the course of the disease, but that this may only be observed infrequently, as CK is not part of the standard peripheral neuropathy work-up.

Our study faced some limitations, including the small sample size and the retrospective study design. Also, we did not perform quantitative EMG for our patients.

In summary, our study provides some evidence that peripheral neuropathy, including the more commonly encountered acquired etiologies, may feature some degree of hyperCKemia, and that peripheral neuropathy patients experiencing cramping may have higher CK levels than their asymptomatic counterparts. It is important to note that this association occurred in the absence of an underlying myopathy or alternate cause of hyperCKemia or cramping. Highlighting this association is essential to avoid unnecessary and invasive testing, such as muscle biopsy, in patients with peripheral neuropathy, except in the appropriate clinical setting, as discussed earlier.

## Data Availability Statement

The raw data supporting the conclusions of this article will be made available by the authors, without undue reservation.

## Ethics Statement

The studies involving human participants were reviewed and approved by the Institutional Review Board at The Ohio State University Wexner Medical Center. Written informed consent for participation was not required for this study in accordance with the national legislation and the institutional requirements.

## Author Contributions

AN, AJ, BE, and WA contributed to the conception and design of the study. AN and AJ organized the database and wrote the first draft of the manuscript. WA performed the statistical analysis. All authors contributed to manuscript revision, read and approved the submitted version.

## Conflict of Interest

JH received compensation for consulting for Reata and Avexis. WA received compensation for consulting for La Hoffmann Roche and Genentech. BE received compensation for consulting for Biogen, Argenx, and Stealth Bio-therapeutics. The remaining authors declare that the research was conducted in the absence of any commercial or financial relationships that could be construed as a potential conflict of interest.
